# Improving drinking water quality through proficiency testing—the impact of testing method and accreditation status on *Escherichia coli* detection by Canadian environmental testing laboratories

**DOI:** 10.3389/fmolb.2024.1338549

**Published:** 2024-05-01

**Authors:** Mahfuza Sreya, Md Saiful Alam, Sahibjot Daula, Caleb Lee, Veronica Restelli, Ken Middlebrook, Michael A. Noble, Lucy A. Perrone

**Affiliations:** ^1^ Canadian Microbiology Proficiency Testing Program (CMPT), Department of Pathology and Laboratory Medicine, University of British Columbia, Vancouver, BC, Canada; ^2^ School of Population and Public Health, University of British Columbia, Vancouver, BC, Canada; ^3^ Department of Chemistry, University of British Columbia, Vancouver, BC, Canada; ^4^ Proficiency Testing Canada (PTC), Ottawa, ON, Canada

**Keywords:** drinking water, proficiency testing, accreditation, external quality assessment, microbial contamination

## Abstract

Water quality testing is crucial for protecting public health, especially considering the number of boil water advisories annually issued across Canada that impact daily life for residents in affected areas. To overcome these challenges, the development of drinking water safety plans and accessibility to regular testing using simple, rapid, and accurate materials are necessary. However, the significance of monitoring the accuracy of environmental microbiology testing laboratories cannot be overlooked. Participation in external quality assessment programs, such as those that include proficiency testing (PT), is a necessary risk management resource that ensures the effectiveness of these testing processes. Proficiency Testing Canada (PTC), in collaboration with the Canadian Microbiological Proficiency Testing (CMPT) program based at the University of British Columbia, have implemented a drinking-water microbiology PT program since 1996. Both PTC and CMPT are ISO/IEC 17043:2010-accredited EQA providers. The drinking water program provided PT challenges to subscribing testing laboratories twice per year. Each challenge consisted of four samples containing unknown concentrations of *Escherichia coli (E. coli)* and *Enterobacter* spp. Results from participants were assessed for accuracy based on the method of testing. This cross-sectional study evaluated 150 rural and metropolitan testing sites across Canada between 2016 and 2022. Multivariable logistic regression analysis was conducted to examine the impact of different testing methods and laboratory accreditation status on the proficiency scores. This approach enabled us to assess the association between multiple independent variables and the likelihood of achieving specific proficiency scores, providing insights into how testing methods and accreditation status affect overall performance. After adjusting for rural residence, testing time, and survey year, the membrane filtration method was positively associated with the likelihood of scoring satisfactory results compared to the enzyme-substrate method (OR: 1.75; CI: 1.37–2.24), as well as accreditation status (OR: 1.47; CI: 1.16–1.85). The potential for improvement in environmental laboratory testing performance through the implementation of regulated PT in drinking water safety plans is proposed, along with the need for reliable testing methods applicable to rapid drinking water microbiology testing.

## 1 Introduction

Accurate quantification of microbial contamination in drinking water is imperative to the decisions made by Canadian provincial public health authorities for issuing public health alerts. Decisions ranging from bans on swimming in local lakes to issuing boil water advisories to entire communities are subject to reliable testing of microbial contamination indicators by regional environmental testing laboratories. Health Canada, which is responsible for issuing national health policy under the Government of Canada, has issued the *Guidance on Monitoring the Biological Stability of Drinking Water in Distribution Systems* (Health Canada, 2022) and *Guidance for providing safe drinking water in areas of federal jurisdiction* (Health Canada, 2021). Both documents outline recommendations for drinking water safety plans based on the World Health Organization’s *Guidelines for Drinking-Water Quality* (WHO, 2017). Although these documents highlight the complexity of a sustainable drinking water monitoring system, both Canadian guidelines fail to emphasize the importance of enrolling in external quality assessment (EQA) as a means to verify the accuracy of the identification and quantification of microorganisms and to monitor the efficacy of the drinking-water safety plan after implementation.

One key pillar of EQA includes enrollment in a proficiency testing (PT) program, and which is also a requirement of laboratory accreditation to ISO/IEC 17025:2017 G*eneral Requirements for the Competence of Testing and Calibration Laboratories*. PT programs send samples containing relevant hazard indicators of known concentration to testing laboratories for blinded analysis. These programs serve as one way to ensure the accuracy and reliability of detecting pathogenic indicators taken from regular drinking water samples, as well as the reporting and subsequent public health measures taken by the responsible laboratories (Molina-Castro et al., 2021). In particular, drinking water microbiology is often monitored by *Escherichia coli* (*E. coli*) detection. *E. coli* is an internationally recognized indicator of fecal contamination in drinking water that is associated with high public risk. *E. coli* is an ideal indicator as it is excreted in high numbers by animals and humans, tends to remain stable in drinking water, and is easily detected in comparison to enteric parasites and viruses (World Health Organization, 2017). Therefore, the presence of *E. coli* in drinking water is an indication of the possible presence of other disease-causing fecal microorganisms of concern. Such detection leads to the release of public health advisories, namely, boil water advisories.

In 2021, 18.4% of the total boil water advisories indicated “no applicable water quality reason” (Environment and Climate Change Canada, 2022). This suggests that reasons beyond the physical infrastructure of the drinking water system were cause for concern and led to the issuing of a public health notice. In Ontario, Canada, ISO/IEC 17025 accreditation and enrollment in PT became mandatory following the Report of the Walkerton Inquiry (O’Connor, 2002). In May 2000, 7 people died and over 2,300 became ill after agricultural runoff containing *E. coli* O157:H7 and *Campylobacter jejuni* entered the drinking water system in the small town of Walkerton, Ontario. An investigation into the incident found that the Walkerton Public Utilities Commission operators did not have the training and expertise to identify potential breaches in contamination, and budget cuts led to irregular monitoring of the drinking water safety system, which led to delayed boil water advisories in the region. Since then, the requirement for mandatory laboratory accreditation was fully integrated into the Ontario Safe Drinking Water Act in 2002 (Ontario, 2016). It was noted that although enrollment in accreditation and, thus, PT programs does not guarantee the accuracy of testing results, it offers a means of external and objective monitoring that has led to direct evaluation of laboratory testing and reporting systems.

Despite the definitive decisions made in Ontario after the Walkerton Inquiry, there is currently no legal requirement for other Canadian environmental testing laboratories to enroll in a PT program or obtain accreditation. The Standards Council of Canada (SCC) was established in 1970 to promote the voluntary adoption of standardized practices. Alongside the SCC, the Canadian Association for Laboratory Accreditation (CALA) and the Centre d’expertise en analyse environmentale du Québec in Quebec provide accreditation services to ISO/IEC 17025 and therefore require enrollment in PT. PT is regularly used internationally to evaluate the efficacy of drinking water testing within a region (Noble and Nikiforuk, 1996; Kelleher et al., 2017; Cao and Yang, 2020; Molina-Castro et al., 2021). Previous literature from CALA has also led to the evaluation of accredited and non-accredited environmental laboratories, where accredited laboratories were found to be more likely to show accurate results in PT (Morris and Macey, 2004; Middlebrook, 2017).

PT is a tool with which organizations can continually assess and monitor their testing process through interlaboratory comparison. This includes monitoring the training of laboratory staff, methodology, and the ability of staff to report accurate findings. This allows laboratories to identify risks within the drinking water quality system before public health notices are needed.

In 2006, PTC, in collaboration with the Canadian Microbiology Proficiency Testing program at the University of British Columbia, launched the “Microbiology in Water” program. Both PT providers are accredited under ISO/IEC 17043:2010 *Conformity assessment—General requirements for the competence of proficiency testing accredited PT providers* (International Organization of Standardization, 2010). This program sends four samples of known concentrations of wild-type *E. coli* for blind analysis by the participating laboratory. These samples are combined with wild-type *Enterobacter* spp. at varying concentrations to emulate the lack of homogeneity in field drinking water samples. These samples are sent two times a year, in March and October, to monitor the ongoing performance of both public health and private testing laboratories that choose to enroll. This PT scheme is designed to be tested according to the environmental laboratory’s typical operation.

Awareness of the efficacy of PT programs as a means of monitoring drinking water systems is lacking (Kelleher et al., 2017; Molina-Castro et al., 2021). Studies evaluating the efficacy of PT program implementation in drinking water do not mention the specificity and complexity of infectious pathogen testing. This study aims to evaluate the efficacy of common methods of *E. coli* quantification and accreditation status and their association with satisfactory proficiency scores in a nationwide study of environmental testing laboratories.

## 2 Materials and methods

### 2.1 EQA program design

PTC and CMPT collaborated in designing the “Microbiology in Water” PT program scheme. The scheme consisted of four different samples per survey. Each survey was sent biannually, typically in March and October of each year. Participants chose to subscribe to one survey or both throughout the year on a fee-for-service basis. Each sample consisted of 5 mL of bacterial stabilizer spiked with known concentrations of live *E. coli* and *Enterobacter* spp. to reflect the heterogeneity of fecal-contaminated field drinking water samples (Table 1). Samples were created and sent on the same day in order to maintain stability. PTC and CMPT recommended to participants that samples be tested within 96 h of the shipment date. Once received, participants diluted 1 mL of the sample into 1,000 mL of sterile distilled water (1:1,000 dilution), which could then be used to test for total coliforms and *E. coli* according to the participant’s established protocols for membrane filtration (MF) using the agar(s) of choice, enzyme-substrate methods (EST), or the most probable number (MPN) method. Target concentrations of samples for each survey year were determined 1 year prior by the CMPT senior technician within a range of 20–100 CFU/100 mL per organism. This was done with careful consideration to avoid repeated testing values from the previous 2 years of surveys.

**TABLE 1:** An example of a Microbiology in Water survey set.

C05A-1 (mL)	C05A-2 (mL)	C05A-3 (mL)	C05A-4 (mL)
*Escherichia coli*: 30 CFU/100	*Escherichia coli*: 50 CFU/100	*Escherichia coli*: 20 CFU/100	*Escherichia coli*: 80 CFU/100
*Enterobacter* spp.: 30 CFU/100	*Enterobacter* spp.: 60 CFU/100	*Enterobacter* spp.: 70 CFU/100	*Enterobacter* spp.: 20 CFU/100

Samples are blinded to participants using the unique survey code. The CMPT senior technician selects the sample concentrations of each vial based on the sample concentrations of the previous year so as to avoid repeated survey challenges.

### 2.2 Sample preparation and validation

Each sample was prepared in single batches to maintain consistency across all survey sets. Bacterial stabilizer was prepared as previously described (Brodsky et al., 1978; Noble and Nikiforuk, 1996). Twenty-four hours before shipment of the survey, the colonies of *E. coli* and *Enterobacter spp*. were inoculated in respective tubes of 10 mL sterile Mueller Hinton broth (Oxoid, Nepean, Canada) and incubated at 37°C in O_2_ overnight to obtain pure cultures of each organism. On the day of the shipment, the bacteria were centrifuged at 1711 *g* for 10 min at room temperature. The pellets were washed with sterile phosphate-buffered saline (PBS) twice, and resuspended in PBS. The initial quantity of each organism was determined by measuring the optical density at 570-nm. Serial dilutions were made in PBS to the previously established target concentration of each sample. The final dilution of each organism was pipetted into the beaker of bacterial stabilizer. Aliquots of 5 mL were dispensed into sterile vials (Sarstedt, Nümbrecht, Germany). The vials were sealed with o-ring caps, parafilmed, and shipped according to the UN3373 guidelines under the Transportation of Dangerous Goods Regulations (Transport Canada, 2022). 10% of samples from each batch were allocated for internal quality control at CMPT through systematic random sampling. Samples were divided and kept at room temperature and at 4°C to be tested at intervals of 24 h, 72 h, and 168 h after shipment. Sample concentrations were diluted as described above and validated for sample homogeneity and stability by MF.

### 2.3 Data collection and PT scoring

Participant results were collected electronically or by facsimile from participants. Data was collected on participant’s location, accreditation status, testing method, date analyzed, and quantified *E. coli* count by CFU/100 mL or MPN/100 mL. The participating laboratories’ names and addresses were deidentified. The data was stratified in Microsoft Excel by the given sample codes: C05A-1, C05A-2, C05A-3, and C05A-4. Then, each sample was stratified by method type to calculate the PT score of each survey. The PT score was calculated using the methods outlined in ISO/IEC 17043 (International Organization of Standardization, 2017). The scoring process consisted of calculating the mean, standard deviation, and z-score of the reported values by each testing method before assigning a PT score.

Outliers were defined as data points of extreme values and were identified visually (e.g., reported values of >1000 CFU/100 mL or MPN/100 mL). The decision to remove outliers visually was based on several careful considerations. Visually excluding data points at the extremities allowed us to directly assess the data in the context of the overall distribution of results and valid *E*. *coli* quantification as a proficiency testing provider. Stringent statistical cutoffs disproportionally removed valid data points that were critical to the nuances of each *E. coli* quantification method. Therefore, we were able to exercise discretion in considering not only the statistical anomalies, but also the relevance of each data point. We acknowledge that this approach can introduce subjectivity and should be used with discretion. To maintain rigor, we involved two independent researchers to score the proficiency testing survey and identify outliers in the process. The number of scored surveys was divided equally between the two researchers prior to analysis.

Starting with C05A-1, the mean (X) and the standard deviation (δ) of the reported values (x) for all testing methods were calculated using Excel. Each sample was given a z-score (z) based on the formula:

$$z=\frac{x\;-\;X}{\delta }$$

Each sample was then manually given a PT-Score based on the z-score criteria (Table 2). The mean, standard deviation, and z-score calculations were then repeated for each individual testing method, followed by manual PT-scoring of each method’s reported values based on the z-scores for the C05A-2, C05A-3 and C05A-4 sample codes.

**TABLE 2:** ISO/IEC 17043:2010 Guidelines for proficiency test scores based on z-score.

Z-Score range	PT-score
|z| < 2	Satisfactory
2 ≤ |z| < 3	Questionable
|z| ≥ 3	Unsatisfactory

### 2.4 Statistical analysis

#### 2.4.1 Variable selection

Statistical analysis in this study was conducted using STATA ver. 17 software (STATACorp, 2021). The distribution of each variable in relation to the outcome variable “Proficiency score” was compared using the Chi-square test, with results expressed as frequencies and percentages. To investigate the association between primary exposure “accreditation status” and “testing method” and the outcome of “proficiency score,” we employed multiple logistic regression analysis for a complete case analysis. Relevant confounding variables, specifically rural residence, as well as risk factors for the outcome (testing time post-shipment and survey year), were included and conceptualized using a directed acyclic graph (DAG) (Supplementary Figure S1). The model was adjusted for the minimal sufficient adjustment set derived from the DAG, which included rural residence. Additionally, risk factors for the outcome, such as testing time post-shipment and survey year, were included in an automated backward stepwise regression with the Akaike Information Criterion (AIC) to enhance the precision of estimates.

#### 2.4.2 Logistic regression modeling

Two model specifications were considered: the upper bound, which incorporated both the minimal sufficient adjustment set and the risk factors for the outcome, and the lower bound, which included only the minimal sufficient adjustment set. Furthermore, we conducted tests to assess the interaction effect of rural residence in relation to exposure and outcome. Once the main effect model was defined, analysis of variance (ANOVA) was employed to evaluate whether the model that included the interaction terms was statistically significant. Model performance was assessed using a Receiver Operating Characteristics (ROC) curve, and goodness of fit was evaluated using the Hosmer-Lemeshow test. Measures of association were reported using odds ratios (OR) along with corresponding confidence intervals (CI). All statistical tests were two-sided, with a significance level set at *p < 0.05*.

## 3 Results

**FIGURE 1:**
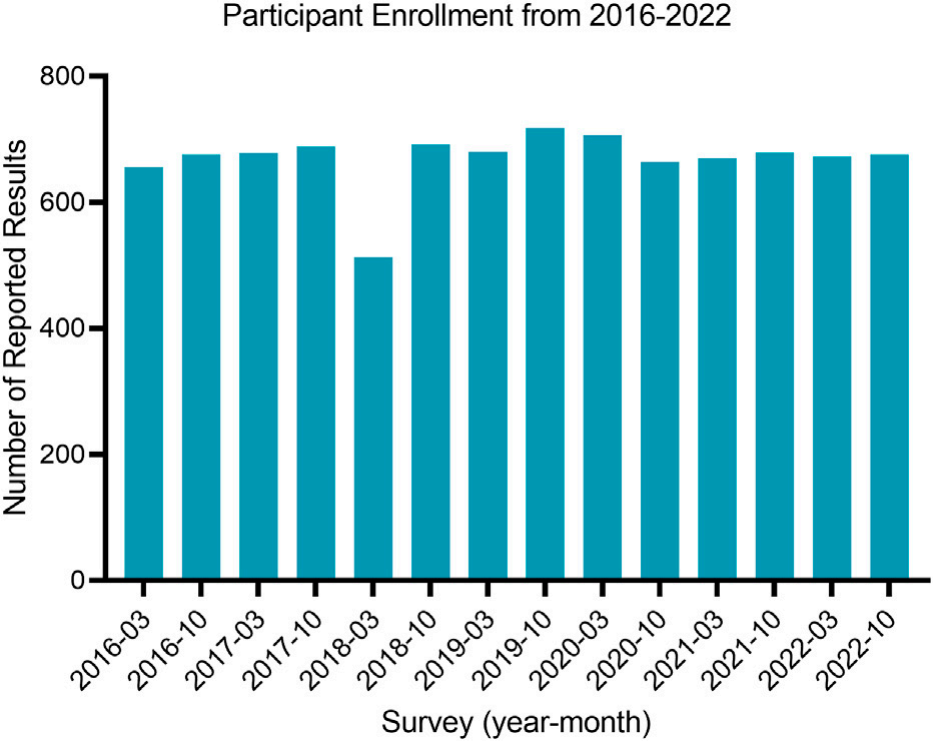
The number of reported results per survey in the “Microbiology in Water” proficiency testing program.

The majority of participants in the study (95.4%) achieved satisfactory proficiency testing (PT) scores, whereas a small proportion (4.6%) attained unsatisfactory PT scores, as presented in Table 3. Enrollment in the “Microbiology in Water” program had a total of 150 Canadian participants with consistent participation in surveys from 2016–2022, with a slight decrease in March of 2018 (Figure 1). The majority of participants were located in metropolitan areas (87.6%), with Ontario making up the highest proportion of participants (40.4%) and Alberta being the second highest (15.4%). The majority of participants reported results using membrane filtration (62.5%) or the enzyme-substrate method (28.3%) and were accredited through CALA (68.0%).

**TABLE 3:** Characteristics of Canadian environment testing laboratories who were enrolled in external quality assessment (EQA) which includes proficiency testing (PT) program from 2016 to 2022 based on the total samples analyzed (N = 9,367).

Variable	Total samples analyzed (%)
Participant Characteristics	
Province	
Alberta	1,442 (15.4)
British Columbia	540 (5.8)
Manitoba	349 (3.7)
New Brunswick	662 (7.1)
Newfoundland and Labrador	214 (2.3)
Nova Scotia	694 (7.4)
Northwest Territories	51 (0.5)
Ontario	3,787 (40.4)
Prince Edward Island	55 (0.6)
Quebec	157 (1.7)
Saskatchewan	495 (5.3)
Yukon	77 (0.8)
Missing	844 (9.0)
Rural	
Yes	8,202 (87.6)
No	321 (3.4)
Missing	844 (9.0)
Testing Characteristics	
Accreditation (CALA)	
Yes	6,373 (68.0)
No	2,994 (32.0)
Testing Method	
Membrane filtration	5,850 (62.5)
Enzyme-substrate	2,653 (28.3)
Most probable number	813 (8.7)
Missing	51 (0.5)
Testing time post-shipment	
Less than or equal to 96 h	6,856 (73.2)
Greater than 96 h	2,513 (26.8)
Overall score	
Satisfactory	8,932 (95.4)
Unsatisfactory	435 (4.6)

Satisfactory performance varied significantly between those using EST (93.8%), MF (96.5%), and MPN (93.6%) methods (*p* < 0.001) and those accredited to ISO/IEC 17043 through CALA (96.2%) (*p* < 0.001) (Table 4). After adjusting for confounding factors (testing time post-shipment, rural status, and survey year), the likelihood of satisfactory results is 1.75 times higher using the MF method compared to the EST method (CI: 1.37, 2.24; *p* < 0.001), and 1.47 times higher when the laboratory is accredited (CI: 1.16, 1.85; *p* < 0.001) compared to non-accredited laboratories (Table 5). In addition, there is a 75% decrease in the likelihood of a satisfactory result in participants that tested 96 h or longer post-shipment (%) (CI: 0.59, 0.98; *p* < 0.05).

**TABLE 4:** Bivariate analysis findings of Canadian environment testing laboratories who were enrolled in external quality assessment (EQA) which includes proficiency testing (PT) program from 2016 to 2022 stratified by satisfactory/unsatisfactory proficiency testing scores (*N* = 9,367).

	Proficiency testing score		
	Satisfactory (*n* = 8,932)	Questionable or unsatisfactory (*n* = 434)	*p*-value
Primary Outcomes			
Testing method			<0.001
EST	2,488 (93.8%)	164 (6.2%)	
MF	5,647 (96.5%)	203 (3.5%)	
MPN	761 (93.6%)	52 (6.4%)	
Accreditation status (CALA)			<0.001
Yes	6,129 (96.2%)	243 (3.8%)	
No	2,803 (93.6%)	191 (6.4%)	
Confounding factors			
Rural location status			<0.001
Yes	313 (97.5%)	8 (2.5%)	
No	7,884 (96.1%)	317 (3.9%)	
Testing time post shipment^a^			<0.001
<96 h	6,589 (96.2%)	263 (3.8%)	
≥96 h	2,240 (93.4%)	158 (6.6%)	
Survey (year)			0.069
2016	1,249 (93.8%)	83 (6.2%)	
2017	1,304 (95.5%)	62 (4.5%)	
2018	1,149 (95.4%)	55 (4.6%)	
2019	1,329 (95.1%)	68 (4.9%)	
2020	1,312 (95.8%)	58 (4.2%)	
2021	1,305 (96.7%)	44 (3.3%)	
2022	1,284 (95.3%)	64 (4.7%)	

^a^ Testing time post-shipment *n* = 9,251.

**TABLE 5:** Crude and adjusted associations between the testing method, accreditation status and satisfactory/unsatisfactory (reference category) proficiency testing scores among Canadian environment testing laboratories who were enrolled in external quality assessment (EQA) which includes proficiency testing (PT) program from 2016–2022.

	Unadjusted odds ratio (95% CI)	Adjusted odds ratio (95% CI)
Testing Method		
EST	1.0 (Ref)	1.0 (Ref)
MF	1.84 (1.49, 2.28)***	1.75 (1.37, 2.24)***
MPN	0.97 (0.70, 1.33)	0.85 (0.58, 1.23)
Accreditation status to ISO/IEC 17025		
No	1.0 (Ref)	1.0 (Ref)
Yes	1.60 (1.31, 1.95)***	1.47 (1.16, 1.85)***
Rural location status		
No	1.0 (Ref)	1.0 (Ref)
Yes	1.54 (0.76, 3.14)	1.85 (0.89, 3.82)
Testing time post shipment		
<96 h	1.0 (Ref)	1.0 (Ref)
≥96 h	0.22 (0.18, 0.26)***	0.76 (0.59, 0.98)*
Year of program participation		
2016	1.0 (Ref)	1.0 (Ref)
2017	1.48 (1.04, 2.11)*	1.78 (1.13, 2.80)*
2018	1.33 (0.93, 1.90)	1.62 (1.03, 2.56)*
2019	1.22 (0.88, 1.71)	1.17 (0.78, 1.74)
2020	1.43 (1.01, 2.02)*	1.46 (0.96, 2.22)
2021	1.87 (1.28, 2.72)**	1.53 (1.01, 2.32)*
2022	1.28 (0.91, 1.81)	1.05 (0.71, 1.54)

*p < 0.05, **p < 0.01, ***p < 0.001. CI, confidence interval.

## 4 Discussion

As approaches such as drinking water safety plans become more widely accepted as a tool to develop a preventative framework in Canada, it is important to consider aspects such as testing methods and enrollment in accreditation, or PT, to improve drinking water quality within the context of individual communities. In this study, 150 Canadian laboratories participated in drinking water microbiology PT from 2016 to 2022, where the likelihood of a participant scoring a satisfactory result was significantly higher when using the MF method compared to the EST (Table 4), regardless of the culture medium used. MF itself proves to be the most prevalent method across Canada (Table 4), which further indicates its validity for reliable *E. coli* quantification. These findings are consistent with the recommendations of the *Standard Methods for the Examination of Water and Wastewater*, which establishes MF as a “gold standard” due to its ability to accurately quantify *E. coli* by colony count and high sensitivity for microorganisms within large volume samples (Rompré et al., 2002; Health Canada, 2020; American Public Health Association American Water Works Association Water Environment Federation, 2023).

The enzyme-substrate method has grown in popularity since its release in the 1990s as a rapid and streamlined MPN quantification method (Gorski et al., 2019). The EST method, namely, the Colilert tests (IDEXX Laboratories, Portland, ME, United States), utilizes the constitutive enzyme β-glucuronidase to detect *E. coli* by blue-white fluorescence due to its previous studies comparing standard MPN and MF methods to EST, which have produced variable results. Studies found that the EST tests generally underestimated *E. coli* recovery and produced a 10%–11% false negative rate (Schets et al., 2002; Fricker et al., 2010). Others comparing standard MPN and MF methods to EST have found that the methods show no significant difference in recovery of *E. coli* or have found EST to be a more sensitive method compared to the MPN method (Eckner, 1998; Kämpher et al., 2008); however, the PT samples used in this study control for confounding factors, such as non-coliforms in drinking water, that may lead to false-positive results.

The variety of EST findings, as indicated in this study, reflect the variability of the EST method as a whole. The accuracy of this can be significantly affected by the expression of β-glucuronidase, where recovery of 74 different fecal and environmental strains of *E. coli* has been found to be as low as 51.4% using the Colilert method (Maheux et al., 2008). In the context of this PT scheme for live *E. coli*, pre-analytical factors such as temperature fluctuations, pressure changes, and storage in a bacterial stabilizer could have affected the *E. coli* recovery by EST by the participants despite controlling for such factors through detailed consideration of the packaging.

Rurally located laboratory participants are more likely to use the EST method compared to the MF or MPN methods. Health Canada has outlined these concerns surrounding the EST method, yet rural communities face the challenge of a lack of trained staff, a lack of guidelines, or monitoring procedures specific to the rural laboratory (Health Canada, 2012; Lane et al., 2018). The EST method is efficient and requires minimal labour to process a sample. However, as communities begin to adopt water safety plans, careful consideration must be given to the risks of using EST, as environmental strains may produce false negative results.

Our study revealed that participants that were accredited to ISO 17025:2017 were more likely to produce a satisfactory score (Table 4). These results are similar to those found in previous studies of performance among accredited laboratories (Morris and Macey, 2004). This may be a reflection of the benefits of ongoing monitoring of the quality management system of the individual laboratory, as regular enrollment in programs such as PT offers the chance for laboratories to reflect and improve not only the testing methods but the overall organization and documentation. However, enrollment in PT on its own can be beneficial. It is an established method for the QA/QC of safe drinking water systems that allows environmental laboratories to demonstrate the capability of accurately quantifying *E. coli* and demonstrate root cause analysis (Root et al., 2014).

The primary strength of this study lies in its robust data source, using a cross-sectional design that spans 6 years of participation. This comprehensive approach involves 150 participating laboratories form various Canadian provinces, enhancing the study’s breadth and representativeness. The participant data was collected in real-time as reported by the participants and PT score allocation was blinded to avoid risk of investigator bias from prior knowledge of the laboratory. The PT score analysis was conducted using ISO/IEC 17043 guidelines in order to reflect the interlaboratory comparison that would be conducted by a PT or accreditation provider. Moreover, the data includes environmental laboratories from all 13 provinces and territories across Canada, which increases the generalizability of the water microbiology results and observations nationwide. However, the data used in this study was not initially conducted for the purpose of analysis beyond each survey. Therefore, there may be additional confounding variables, such as technician skills and training, variation among method protocols, and participants’ reporting, that could not be controlled for. Basing the analysis on participant reported results may lead to low confidence, as method protocols within each subgroup may vary drastically and methods reported at MPN may indicate the use of EST instead. As well, in order to evaluate a true positive correlation between continued participation in PT or accreditation and a satisfactory test result, the study would need to be repeated to look at results from particular laboratories beyond 2016 to evaluate the performance of selected laboratories over time. However, the nature of PT programs in drinking water microbiology is currently voluntary; therefore, it is difficult to follow laboratories continuously.

## 5 Conclusion

The MF method is more likely to produce satisfactory results in comparison to the EST method for the measurement of *E. coli* in drinking water. The variability in EST methods detected through PT participation calls for the need to develop efficient and reliable methods to quantify *E. coli* that can be used by laboratories regardless of geographic location. PT itself offers laboratories an opportunity to improve their general standards of practice, testing methods, and quality management.

## Data Availability

The original contributions presented in the study are included in the article/Supplementary Material, further inquiries can be directed to the corresponding author.

## References

[B1] American Public Health Association American Water Works Association Water Environment Federation (2023). Standard methods for the examination of water and wastewater. Washington: APHA Press.

[B2] Brodsky M. H. Ciebin B. W. Schiemann D. A. (1978). Simple bacterial preservation medium and its application to proficiency testing in water bacteriology. Appl. Environ. Microbiol. 35, 487–491. 10.1128/aem.35.3.487-491.1978 25046 PMC242867

[B3] Cao N. Yang J. (2020). Proficiency test for determination of chlorate in drinking water. Wei Sheng Yen Chiu 49, 630–634. 10.19813/j.cnki.weishengyanjiu.2020.04.020 32928360

[B4] Eckner K. F. (1998). Comparison of membrane filtration and multiple-tube fermentation by the colilert and enterolert methods for detection of waterborne coliform bacteria, *Escherichia coli*, and enterococci used in drinking and bathing water quality monitoring in southern Sweden. Appl. Environ. Microbiol. 64, 3079–3083. 10.1128/AEM.64.8.3079-3083.1998 9687478 PMC106820

[B5] Environment and Climate Change Canada (2022). Canadian environmental sustainability indicators: boil water advisories . www.canada.ca/en/environment-climate-change/services/environmental-indicators/boil-wateradvisories.html (Accessed August 28, 2023).

[B6] Fricker C. R. Warden P. S. Eldred B. J. (2010). Understanding the cause of false negative beta-D-glucuronidase reactions in culture media containing fermentable carbohydrate. Lett. Appl. Microbiol. 50, 547–551. 10.1111/j.1472-765X.2010.02834.x 20374452

[B7] Gorski L. Rivadeneira P. Cooley M. B. (2019). New strategies for the enumeration of enteric pathogens in water. Environ. Microbiol. Rep. 11, 765–776. 10.1111/1758-2229.12786 31342654

[B8] Health Canada (2012). Guidance on monitoring the biological stability of drinking water in distribution systems . https://www.canada.ca/en/health-canada/services/publications/healthy-living/guidance-monitoring-biological-stability-drinking-water-distribution-systems.html (Accessed August 28, 2023).

[B9] Health Canada (2021). Guidance on monitoring the biological stability of drinking water in areas of federal jurisdiction . https://www.canada.ca/en/health-canada/services/publications/healthy-living/guidelines-canadian-drinking-water-quality-guideline-technical-document-escherichia-coli.html (Accessed August 28, 2023).

[B10] Health Canada (2022). Guidelines for Canadian drinking water quality: guideline technical document – *Escherichia coli* . https://www.canada.ca/en/health-canada/services/publications/healthy-living/guidelines-canadian-drinking-water-quality-guideline-technical-document-escherichia-coli.html (Accessed August 28, 2023).

[B11] International Organization for Standardization [ISO] (2010). Conformity assessment — general requirements for proficiency testing. [ISO/IEC 17043:2010]. Geneva: International Organization for Standardization.

[B12] International Organization for Standardization [ISO] (2017). General requirements for the competence of testing and calibration laboratories. [ISO/IEC 17025:2017]. Geneva: International Organization for Standardization.

[B13] Kämpfer P. Nienhüser A. Packroff G. Wernicke F. Mehling A. Nixdorf K. (2008). Molecular identification of coliform bacteria isolated from drinking water reservoirs with traditional methods and the Colilert-18 system. Int. J. Hyg. Environ. Health 211, 374–384. 10.1016/j.ijheh.2007.07.021 17870668

[B14] Kelleher K. Wong J. Leon-Vintro L. Currivan L. (2017). International Rn-222 in drinking water interlaboratory comparison. Appl. Radiat. Isot. 126, 270–272. 10.1016/j.apradiso.2017.01.036 28187931

[B15] Lane K. Stoddart A. K. Gagnon G. A. (2018). Water safety plans as a tool for drinking water regulatory frameworks in Arctic communities. Environ. Sci. Pollut. Res. Int. 25, 32988–33000. 10.1007/s11356-017-9618-9 28710728

[B16] Maheux A. F. Huppé V. Boissinot M. Picard F. J. Bissonnette L. Bernier J.-L. T. (2008). Analytical limits of four beta-glucuronidase and beta-galactosidase-based commercial culture methods used to detect *Escherichia coli* and total coliforms. J. Microbiol. Methods 75, 506–514. 10.1016/j.mimet.2008.08.001 18760312

[B17] Microsoft Corporation (2018). Microsoft Excel. Available at: https://office.microsoft.com/excel.

[B18] Middlebrook K. (2017). Do accredited laboratories perform better in proficiency testing than non-accredited laboratories? Accredit. Qual. Assur 22, 111–117. 10.1007/s00769-017-1262-z

[B19] Molina-Castro G. Venegas-Padilla J. Molina-Marcia J. Scarioni L. Calderon-Jimenez B. (2021). Improving the quality control of drinking water in Nicaragua through proficiency testing in a metrological multilateral cooperation project. Sci. Rep. 11, 16853. 10.1038/s41598-021-96230-w 34413351 PMC8377044

[B20] Morris A. Macey D. (2004). Laboratory accreditation: proof of performance for environmental laboratories—2001 study. Accredit. Qual. Assur 9, 52–54. 10.1007/s00769-003-0736-3

[B21] Noble M. A. Nikiforuk S. (1996). Variability in urine culture reporting by Canadian microbiology laboratories. Can. J. Infect. Dis. 7, 247–249. 10.1155/1996/238498 22514446 PMC3327405

[B22] O’Connor D. R. (2002). Report of the Walkerton Inquiry: the events of may 2000 and related issues: a summary. Toronto: Ontario Ministry of the Attorney General.

[B23] Rompré A. Servais P. Baudart J. de-Roubin M. R. Laurent P. (2002). Detection and enumeration of coliforms in drinking water: current methods and emerging approaches. J. Microbiol. Methods 49, 31–54. 10.1016/s0167-7012(01)00351-7 11777581

[B24] Root P. Hunt M. Fjeld K. Kundrat L. (2014). Microbiological water methods: quality control measures for federal clean water Act and safe drinking water Act regulatory compliance. J. AOAC Int. 97, 567–572. 10.5740/jaoacint.13-262 24830168

[B25] Saxena T. Kaushik P. Krishna Mohan M. (2015). Prevalence of *E. coli* O157:H7 in water sources: an overview on associated diseases, outbreaks and detection methods. Diagn Microbiol. Infect. Dis. 82, 249–264. 10.1016/j.diagmicrobio.2015.03.015 26050932

[B26] Schets F. M. Nobel P. J. Strating S. Mooijman K. A. Engels G. B. Brouwer A. (2002). EU Drinking Water Directive reference methods for enumeration of total coliforms and *Escherichia coli* compared with alternative methods. Lett. Appl. Microbiol. 34, 227–231. 10.1046/j.1472-765x.2002.01075.x 11874547

[B27] StataCorp (2021). Stata statistical software: release 17. College Station, TX: StataCorp LLC.

[B28] Transport Canada (2022). Shipping infectious substances . https://tc.canada.ca/en/dangerous-goods/safety-awareness-materials-faq/industry/shipping-infectious-substances (Accessed August 30, 2023).

[B29] World Health Organization (2017). Guidelines for drinking-water quality. Geneva: World Health Organization.

