# Supplementary material for: Improving drinking water quality through proficiency testing—the impact of testing method and accreditation status on Escherichia coli detection by Canadian environmental testing laboratories
Source: Front Mol Biosci. 2024 May 1;11:1338549. doi: 10.3389/fmolb.2024.1338549 (PMC11097683; doi:10.3389/fmolb.2024.1338549)

Supplementary Material

1. Supplementary Figures

Supplementary Figure 1. A directed acyclic graph (DAG) illustrating the minimally sufficient confounding adjustment sets in the relationship between the "accreditation status" and "testing method" and the "proficiency score" among environmental testing laboratories from 2016 to 2022 for multiple logistical regression analysis.

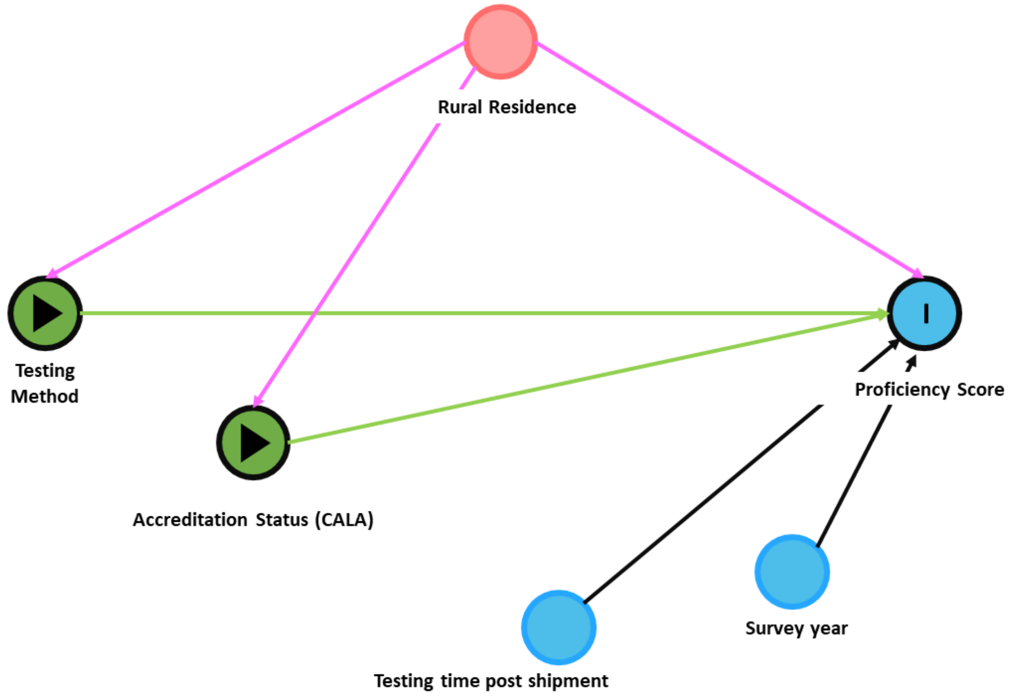

Supplement: Supplementary file 1 [file DataSheet1.pdf]
